# A Novel Bacteriocin Against *Shigella flexneri* From *Lactiplantibacillus plantarum* Isolated From Tilapia Intestine: Purification, Antibacterial Properties and Antibiofilm Activity

**DOI:** 10.3389/fmicb.2021.779315

**Published:** 2022-01-05

**Authors:** Yu-Hang Jiang, Wei-Gang Xin, Qi-Lin Zhang, Lian-Bing Lin, Xian-Yu Deng

**Affiliations:** Faculty of Life Science and Technology, Kunming University of Science and Technology, Kunming, China

**Keywords:** tilapia, *Lactiplantibacillus plantarum*, bacteriocin, *Shigella flexneri*, antibiofilm properties

## Abstract

Few bacteriocins with antibacterial activity against *Shigella flexneri* have been reported. Here, a novel bacteriocin (*LFX01*) produced by *Lactiplantibacillus plantarum* strain LF-8 from the intestine of tilapia was purified and extensively characterized. *LFX01* possesses a molecular weight of 1049.56 Da and an amino acid sequence of I-T-G-G-P-A-V-V-H-Q-A. *LFX01* significantly inhibited *S. flexneri* strain 14 (*S. flexneri*_14) growth. Moreover, it exhibited excellent stability under heat and acid-base stress, and presented sensitivity to a variety of proteases, such as proteinase K, pepsin, and trypsin. The minimum inhibitory concentration (MIC) of *LFX01* against *S. flexneri*_14 was 12.65 μg/mL, which was smaller than that of most of the previously found bacteriocins. Furthermore, *LFX01* significantly inhibited (*p* < 0.05) *S. flexneri*_14 cells and decreased their cell viability. In addition, *LFX01* could significantly (*p* < 0.05) inhibit biofilm formation of *S. flexneri*_14. Scanning electron microscopy analysis presented that the cell membrane permeability of *S. flexneri*_14 was demolished by *LFX01*, leading to cytoplasmic contents leakage and cell rupture death. In summary, a novel bacteriocin of lactic acid bacteria (LAB) was found, which could effectively control *S. flexneri* in both planktonic and biofilm states.

## Introduction

*Shigella flexneri* is a Gram-negative, non-spore-bearing facultative anaerobic bacterium that can cause serious gastrointestinal infections (food poisoning), especially bacillary dysentery (Cui et al., 2015; Jabbar and Al-azawi, 2020). It is also widely known as the “first killer” in the foodborne pathogenic bacteria in children in developing countries (Cui et al., 2015; Nisa et al., 2020). Previous studies reported an estimated 165 million cases of shigellosis worldwide each year, with approximately 1.1 million deaths accounting for 69% of the cases in the immunocompromised 5-year-olds (Mardaneh et al., 2013; Saima et al., 2018). Additionally, *S. flexneri* infecting an animal body could induce microfold cell transportation in animal intestinal mucosa by entering the lymphatic tissues through endocytosis (Ranganathan et al., 2019; Rey et al., 2020). Although macrophages can swallow *S. flexneri*, it can escape by binding *S. flexneri* IpaB with caspase-1 and activate caspase pathways in the host intestinal cells through lysis (Ranganathan et al., 2019; Li L.H. et al., 2020). Thus, preventing and controlling the *S. flexneri* that induced foodborne pathogen infection has become the primary concern of the researchers. *S. flexneri* strains primarily contaminate many high nutritional foods such as fish and poultry, which affect food quality, resulting in human food safety problems (Paul et al., 2019). At present, the primary way to control *S. flexneri* in food is via the use of unhealthful chemical preservatives. Therefore, exploring new methods to control foodborne *S. flexneri* is necessary.

Biofilm is a community of bacteria attached to surfaces of biotic (foods) or abiotic (processing equipment) (Lindsay and Von, 2006; Shahin et al., 2019). Biofilm of foodborne pathogenic bacteria possesses resistance to disinfectants and antibacterial agents, with high resistance to heat and drying. Furthermore, high-density structure of the biofilm protects pathogenic bacteria from acidic and antibiotic treatments (Tsukatani et al., 2020). In particular, cells of pathogenic bacteria in biofilms are 10–1,000 times less sensitive to disinfectants than planktonic cells (Li et al., 2021; Xiang et al., 2021). Moreover, biofilm is one of the primary causes of equipment damage, thus leading to increased energy cost, as well as is involved in the occurrence of food spoilage and foodborne diseases (Nové et al., 2020; Tsukatani et al., 2020). Currently, biofilm formed by pathogenic bacteria and biofilm mediated foodborne disease prevalence has become one of the major challenges for food safety. To date, several studies have explored the biofilm formation ability and mechanism of *S. flexneri* in foods, and employed high-concentration antibiotics and bacteriostatic synthetic compounds to control *S. flexneri* biofilm (Kang et al., 2020; Kaoukab et al., 2020). However, no such effective biological control approaches to inhibit planktonic cells and biofilm of *S. flexneri* have been reported so far.

Bacteriocin from bacteria is a class of natural macromolecular protein or small molecule with short peptide (Li et al., 2017; Enriqueta et al., 2020; Peng et al., 2021). It is also known to be a class of natural antimicrobial agents that can effectively inhibit or inactivate many foodborne pathogenic bacteria (Guo et al., 2020; Darvishi et al., 2021). In particular, many lactic acid bacteria (LAB) bacteriocins have been demonstrated with high safety and tolerance to heat, acids, and bases (Guo et al., 2020). For instance, LAB bacteriocins derived from *Lactococcus lactis* 2Mt and *Lactobacillus salivarius* CGMCC20700 were found to effectively inhibit foodborne *Staphylococcus aureus* under a wide range of acid-base and temperature (Pmka et al., 2019; Li et al., 2021). Although many LAB bacteriocins have been identified so far, their effects on biofilm formation of foodborne *S. flexneri* are still unexplored.

*Lactiplantibacillus plantarum* (previously named as *Lactobacillus plantarum*; Zheng et al., 2020) is a potential probiotic additive (Bu et al., 2021) and is abundant in animal gastrointestinal tracts and feces (Lei et al., 2020), such as bacteriocins produced by swun5815 strain from yak intestine (Zhou et al., 2018), bacteriocin ZJ316 from healthy infant feces (Chen et al., 2018), and bacteriocin *zrx03* from newborn children feces (Lei et al., 2020). *L. plantarum* bacteriocins and other LAB bacteriocins are mostly isolated from the mammalian intestine, but bacteriocins isolated from other animal groups, such as fish are extremely rare. Therefore, the limited bacteriocin resources have also limited the application of LAB bacteriocins.

As the second most commonly cultured freshwater fish worldwide, tilapia aquaculture has been extensively conducted in more than 100 countries, including China, Indonesia, and Egypt (Subasinghe, 2017). Previous studies have isolated and identified bacteriocin-producing LAB strains from the intestine of *Oreochromis niloticus* in Cameroon, Africa (Pmka et al., 2019). However, these studies only obtained crude LAB bacteriocins from tilapia by chloroform/methanol method, and its molecular weight was roughly checked by tricine-SDS-PAGE (Pmka et al., 2019). This evidence implied that intestine of tilapia is an important source of LAB bacteriocin resources, and it likely contains LAB bacteriocins with antibacterial activity against *S. flexneri*. In addition, further purification and characterization (e.g., molecular weight, amino acid sequence composition, antibacterial, and antibiofilm activity) of LAB bacteriocins from tilapia is highly necessitated.

The present study sought to screen the *L. plantarum* strains with high antibacterial activity against *S. flexneri* from tilapia intestines. Subsequently, the *L. plantarum*-producing bacteriocin was purified and its molecular weight and amino acid sequence were determined. Furthermore, the minimum inhibitory concentration (MIC), time-kill kinetics, acid-base, thermal stability, and enzyme sensitivity of bacteriocin were assessed. The inhibitory effects of *LFX01* on *S. flexneris* biofilm were investigated.

## Materials and Methods

### Isolation and Culture of Strain

Healthy juvenile tilapia (*Oreochromis niloticus*) samples were purchased from the food market located in Chenggong District, Kunming, Yunnan, China, and sent to the laboratory immediately within 3–5 h. Later, six individuals (mean body weight: 12 ± 1.5 g, mean body length: 7 ± 1.2 cm) were anesthetized with ethyl 3-aminobenzoate methane sulfonate (concentration: 15 mg/L, MS-222, Sigma, MO, United States). Then, the tilapia individuals were washed twice with 75% ethanol and 5 times with sterile water, and the intestines of tilapia were aseptically cut on a super-clean worktable (Optec, Chongqing, China) and placed in a 5.0-mL sterile Eppendorf tube with 2.0-mL sterile water, and thoroughly ground with a grinding rod. Afterward, 100 μL dilutions of four concentrations (i.e., 10^–1^, 10^–3^, 10^–5^, and 10^–7^) obtained by gradient dilution with sterile water were dispersed on the surface of MRS Culture Medium (Solarbio, Beijing, China) (37°C, 24 h). Furthermore, single colonies were picked out by sterile inoculation loop. Finally, these single colonies were preliminarily identified and stored at 4°C according to their phenotypic characteristics.

### Screening and Identification of Bacteria With Antibacterial Activity

*S. flexneri* strain BDS14 (referred to as *S. flexneri_14*) isolated from chicken was obtained in our previous studies (He et al., 2021), and it is being stored at Faculty of Life Science and Technology, Kunming University of Science and Technology. Species of LAB strains were confirmed by morphological and microscopic observation under a light microscopy (400 × magnification) (Zeiss Primo Star, Jena, Germany) according to the “manual for systematic identification of common bacteria” (Goodfellow et al., 2012). LAB strains possess several morphological characteristics such as rod-shaped appearance, gram-positive stain, no flagella, and no capsule.

The candidate LAB strains were cultured in MRS liquid medium (37°C, 24 h) and the culture solution was centrifuged (7,104 × *g*, 10 min) to discard the precipitate. Subsequently, a 0.22-μm filter membrane was employed to obtain a cell-free suspension. The obtained cell-free supernatant was adjusted to neutral pH (6.5) to exclude the interference of organic acids (including lactic acid). Screening of LAB strains against *S. flexneri*_14 was performed by the Oxford cup (diameter 8.0 mm, sample volume 200 μL) double-layer plate method (Voulgari et al., 2010). In brief, 100 μL of a suspension of *S. flexneri*_14 (10^7^ CFU/mL) cells in exponential phase was spread on the surface of LB semisolid medium (Solarbio). The Oxford cup was placed onto the surface of the LB semisolid medium and 200 μL of the cell-free supernatant was added, cultivated at 37°C for 24 h. Finally, a vernier caliper was used to determine the clearing zone around the well which was a result of the antibacterial activity of the LAB supernatant. Sterile water was used as control and three replicates were performed.

Based on the above obtained inhibitory zone, the LAB strain showing the best antibacterial effects against *S. flexneri*_14 was selected for the downstream experiments. The target strain was added to MRS liquid medium and then incubated at 37°C for 24 h. The taxonomic status of the target strain was verified using molecular markers. Briefly, DNA extraction of the target LAB strain was performed according to the directions of DNA Purification Kit (Tiangen, Beijing, China). PCR reaction system: 2 × Mix 12.5 μL, DNA template 1 μL, each universal primer (8F and 1492R for the bacterial 16S rRNA gene) 0.5 μL. PCR reaction conditions: 94°C for 3 min, 94°C for 32 s, 52°C for 32 s, 72°C for 50 s, final extension at 72°C for 5 min after 30 cycles. The PCR amplicons were detected by 1.5% agarose electrophoresis, and Sanger sequencing was performed by Geneseed Biotech Co., Ltd. (Guangzhou, China). The obtained 16S DNA sequences were aligned into the NCBI database using the online BLAST. MEGA6 software was used to reconstruct a phylogenetic tree (Tamura et al., 2013).

### Purification and Determination of Bacteriocin

The target strain (*L. plantarum*) against *S. flexneri*_14 was added to MRS liquid medium (37°C, 24 h), and then centrifuged (7,104 × *g*, 10 min) to remove cell precipitate. The obtained cell-free supernatant was purified by an ÄKTA purifier automatic chromatograph in tandem coupled with a Superdex™ 30 Increase 10/300 GL Exclusion Chromatography columns (GE Healthcare, Marlborough, United States), as previously described (Li Z.R. et al., 2020; Li et al., 2021). The corresponding substances under each eluted peak were separately collected into a 25-mL centrifuge tube, and their antibacterial activity was determined by the Oxford cup double-plate method. The collection that showed the highest antibacterial activity was then loaded and purified again to obtain a purified antibacterial active substance (named as *LFX01*). Finally, the *LFX01* fraction was preserved by freeze-drying in a FD-2A freeze dryer (Biocoll, Beijing, China), for downstream experiments.

The molecular weight and amino acid sequence of the purified *LFX01* were determined by a Nano LC-MS/MS system, as previously described (Li Z.R. et al., 2020; Li et al., 2021). Briefly, *LFX01* were eluted with 0.1% formic acid (Macklin, Shanghai, China), 100% water and 0.1% formic acid, and 100% acetonitrile. The LC linear gradient elution was implemented: from 6 to 9% B for 6 min; from 9 to 50% B for 30 min; and from 50 to 95% B for 3 min and 95–95% B for 5 min, with a flow rate at 0.3 μL/min. The primary mass spectrum was obtained by a single full scan (MS), and the secondary mass spectrum data is obtained by step-normalized collision energy. The obtained mass spectra signals by MS scan were further assessed for protein identification in Peaks Studio X (Bioinformatics, Waterloo, Canada). Antimicrobial peptide databases (i.e., NCBI, UniProt, and Swiss-Prot) were searched amino acid sequence of bacteriocin using the Blast online server. Finally, the antibacterial effects of *LFX01* were verified by the Oxford cup double-plate method.

### Determination of Bacteriocin Concentration

Bacteriocin concentration was estimated by the Pierce^®^ BCA Protein quantification Kit (Thermo Fisher Scientific, Waltham, United States), according to user instruction. Briefly, bovine serum albumin (BSA) standards (2.0 mg/mL) were diluted in ampoules to prepare the required concentration of the standard solution. Subsequently, the standard solution (25 μL) and the equivalent amount of *LFX01* were pipetted into a 96-well plate. A total of 200 μL BCA working reagent was added to each well, the content was thoroughly mixed for 1 min, incubated room temperature for 25 min. Absorbance of the solution was measured at 565 nm at room temperature using a microplate reader (MR-96A, Mindray, Shenzhen, China). Free-BSA samples were set as control. The concentration of *LFX01* in the samples was measured by plotting a standard curve according to y = 1.058x+0.1164, *R*^2^ = 0.9962.

### Enzyme Sensitivity Test

*LFX01* (1.0 mg/mL) was added separately to catalase and different protease solutions [i.e., pepsin (20 mM in a in PBS, pH 2), trypsin, proteinase K, papain (20 mM in PBS, pH 7.5), and alkaline protease (20 mM in PBS, pH 9.0)] with a final concentration of 1.0 mg/mL (Solarbio). The mixture of *LFX01* and enzyme was treated (37°C, 2 h), followed by deactivation (80°C, 5 min). Enzyme without *LFX01* was set as control. The effect of each different enzyme on the antibacterial activity of *LFX01* was confirmed by the Oxford cup double-plate method. Each experimental sample was independently tested in triplicate.

### Acid-Base and Thermal Stability Test

The following tests were performed to detect the acid-base and thermal stability of *LFX01* (1.0 mg/mL), as previously described (Yi et al., 2018; Zhang et al., 2019): (1) treatment at 37, 60, 80, 100, and 121°C for 30 min; (2) pH of *LFX01* was adjusted to 2.0, 4.0, 6.0, 8.0, 10.0, and 12.0 by 1.0 moL/L HCl or NaOH solution and then treated at 37°C for 2 h. Finally, pH of *LFX01* solution was adjusted back to neutral pH (6.5). The antibacterial activity of *LFX01* against *S. flexneri_*14 under different pH values and temperature was separately assessed by the Oxford cup double-plate method. *LFX01* under room temperature (25°C) and neutral pH value was set as control. Each experimental sample was independently tested in triplicate.

### Minimum Inhibitory Concentration and Time-Kill Kinetics

Determination of the MIC values of *LFX01* against *S. flexneri*_14 was conducted, as previously described (Zhang et al., 2019; Li et al., 2021). Briefly, *S. flexneri*_14 was precultured in LB liquid medium by incubation (37°C for 12 h). Then, *LFX01* (1.0 mg/mL) was serially diluted into PBS. These dilutions (10 μL) were added separately into 90 μL of LB liquid medium with *S. flexneri*_14 (final concentration: 10^7^ CFU/mL), incubated at 37°C in a 96-well plate for 24 h. Growth of *S. flexneri*_14 was assessed in triplicate using a microplate reader at 595 nm. MIC was the lowest concentration of antibacterial substances that completely inhibited growth of *S. flexneri_*14, as previously described (Yi et al., 2018; Li et al., 2021). 2 × MIC was used as the effective concentration for bacteriostatic treatment, as previously reported (Yi et al., 2018; Lanhua et al., 2020).

The time-killing kinetics test was conducted to assess the dynamic changes in killing effects of *LFX01* against the indicator strain across various treatment times. Namely, *LFX01* was mixed with *S. flexneri*_14 (10^7^ CFU/mL) to reach a final concentration (2 × MIC) of *LFX01* and then incubated for 0, 0.5, 1, 1.5, and 2 h. Subsequently, 100 μL of *LFX01* at each incubation time points were spread on LB solid medium by 10 fold serial dilution method. After incubating at 37°C for 24 h, 30∼300 single colonies of *S. flexneri*_14 were selected for counting. *LFX01-*free samples were set as control groups. Each experimental sample was independently tested in triplicate.

### Cell Proliferation and Viability Assay

The metabolic activity of *S. flexneri*_14 cells was determined by the XTT (i.e., 2,3-BIS (2-methoxy-4-nitro-5-sulfonyl)-2H-tetrazo-5-carboxyl) assay kit (Abcam, Cambridge, United Kingdom), according to use instruction strictly. Briefly, planktonic *S. flexneri_*14 samples and 10 μL of *LFX01* (final concentrations: 2 × MIC) were added into a 96-well plate containing 80 μL of LB liquid medium, followed by the mixing and incubated (37°C, 1 h). Subsequently, 10 μL of XTT solution was added, mixed for 30 s and incubated (37°C, 2 h). Following a gentle homogenization of the microplate, absorbance of samples was measured using a microplate reader at 450 nm. Each experimental sample was independently tested in triplicate.

The survival rate of planktonic *S. flexneri*_14 cells after exposure to *LFX01* was assessed by the Cell-Check™ viability/cytotoxicity kit (ABP Biosciences, Rockville, United States). Briefly, planktonic *S. flexneri*_14 cells were treated with *LFX01* (2 × MIC) at 37°C for 10 min. Later, 3 μL of mixture composed of fluorescent dyes NucView Green and propidium iodide (PI) was added to the bacterial suspension, followed by incubated at 37°C for 10 min. *LFX01-*free planktonic *S. flexneri*_14 cells were set as control. The experimental samples were detected on the slides using fluorescence microscopy (Boschida, Shenzhen, China) (200 magnification). Each experimental sample was independently tested in triplicate.

### SEM Analysis

Three milliliters of *S. flexneri*_14 (10^7^ CFU/mL) were centrifuged (7,104 × *g*, 5 min) to collect the precipitated bacterial cells. The bacterial cells were washed thrice with PBS and incubated (37°C, 2 h) with 1 mL of *LFX01* (2 × MIC). Subsequently, the *LFX01* treated bacterial cells were then fixed with 2.5% glutaraldehyde at 4°C for 8 h and dehydrated in the concentration gradient of ethanol (20, 40, 60, 80, and 95%) for 30 min each. Afterward, all samples were transferred to polished silicon wafers (10 × 10 mm) and dried at room temperature (25°C). The samples covered by gold powder were imaged with an SEM (S-3000N, Hitachi, Tokyo, Japan). *LFX01-*free *S. flexneri*_14 was set as control, and each treatment was performed in three replicates.

### Determination of Antibiofilm Activity

*LFX01* was mixed with LB liquid medium and transferred to a 96-well plate to assess the effects of *LFX01* exposure on *S. flexneri*_14 biofilm formation at final concentrations of: *LFX01*-free controls, 1/2 × MIC, 1 × MIC, 2 × MIC. Later, 100 μL of cell suspension of *S. flexneri*_14 was added to these *LFX01* dilutions at different concentrations. After incubation at 37°C for 24 h (the optimal temperature and time for biofilm formation of *S. flexneri*_14), the liquid medium was discarded to remove planktonic *S. flexneri*_14 cells and expose the biofilm. The plates washing wells using PBS buffer, a MycoLight™ Live Bacteria Fluorescence Imaging Kit (AAT Bioquest, CA, United States) was employed to stain the exposed biofilm following the product manuals. Excessive dye was removed using PBS buffer. The formed biofilms were detected with fluorescence microscopy (100 magnification). PBS buffer was added to each well followed by homogenization. Absorbance (OD_595_) of the mixture was determined in a microplate reader. Samples not treated with *LFX01* were used as control.

### Data Analysis

Data analysis was conducted, as previously described (Yi et al., 2018; Li et al., 2021). In brief, results were exhibited as mean ± standard deviation (SD) of independent replicates. IBM^®^ SPSS^®^ Statistics version 22 was used to calculate the significance between two groups employing a non-paired two-tailed *t*-test. One-way ANOVA test was conducted to evaluate significance levels among groups. *P*-values < 0.05 indicated significant statistical difference.

## Results

### Screening of the Target Bacteriocin-Producing Lactic Acid Bacteria

**FIGURE 1:**
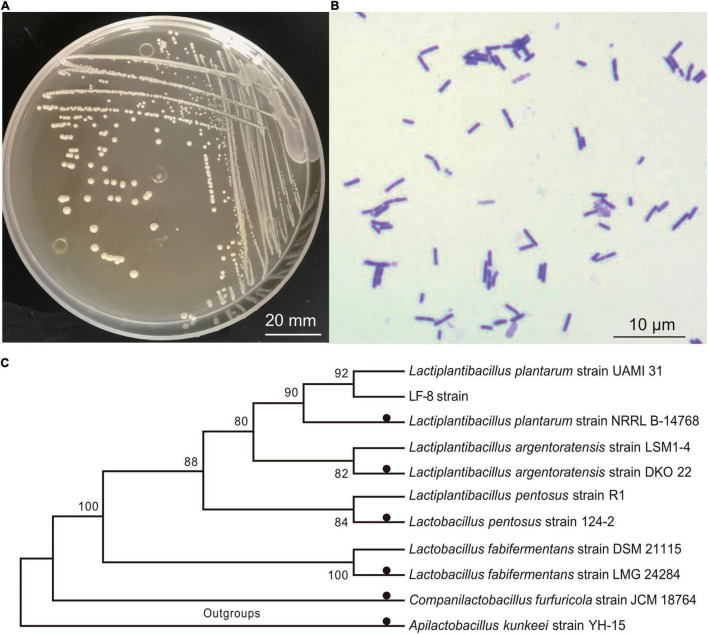
Identification of *Lactiplantibacillus plantarum.* Colony morphology **(A)** and microscopic characteristics; **(B)** following Gram staining **(C)**; phylogenetic analysis of *L. plantarum* LF-8 from 16 rRNA nucleotide sequences. All sequences originated from Lactobacillaceae family and other non-*Lactobacillus* species were used as outgroups. Type strains were marked by solid black circles, and each species of *Lactiplantibacillus/Lactobacillus* included an additional strain.

Among pure cultures of LAB strains isolated from the tilapia intestine, the inhibitory zone diameter of the LF-8 strain against *S. flexneri*_14 was the largest (up to 26.69 ± 0.32 mm). Thus, this strain was selected as the object for further study. Colony morphology and micro-structure of LF-8 revealed white globular, smooth surface, short chain globular (Figures 1A,B). Furthermore, 16S rRNA amplicon of LF-8 with a nucleotide sequence of 1,465 bp (Genbank accession No. MW979597.1) was obtained. BLAST alignment results elucidated a 100.0% similarity of the sequence with *L. plantarum* species (Genbank No. NR117813.1), and the phylogenetic tree presented a cluster of LF-8 strain with *L. plantarum* (Figure 1C). Based on the above-obtained phenotypic and molecular evidence, LF-8 was identified as *L. plantarum*.

### Molecular Weight and Amino Acid Sequence of *LFX01*

**FIGURE 2:**
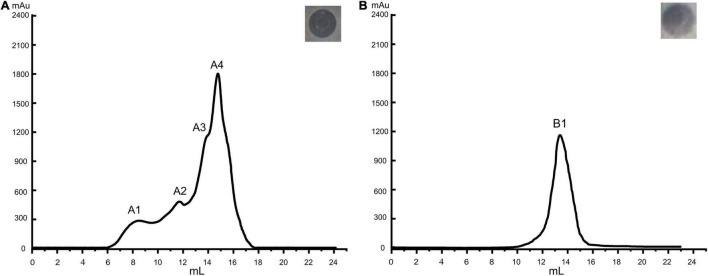
Purification of antibacterial substances produced by *L. plantarum* LF-8. Base peak chromatograms of **(A)** initially purification and **(B)** further purified bacteriocin extract accompanied by the inhibition zones of the corresponding peak against *S. flexneri*_14 as assessed by the Oxford cup double-plate method.

Among the four peaks identified in the chromatogram of the preliminary purified extract, peak A4 showed the greatest antibacterial activity (Figure 2A). The corresponding product in B1 peak was collected after purifying A4 and the antibacterial activity of B1 substances (*LFX01*) against *S. flexneri*_14 was confirmed by the formation of an inhibitory zone (25.12 ± 0.16 mm) (Figure 2B).

**FIGURE 3:**
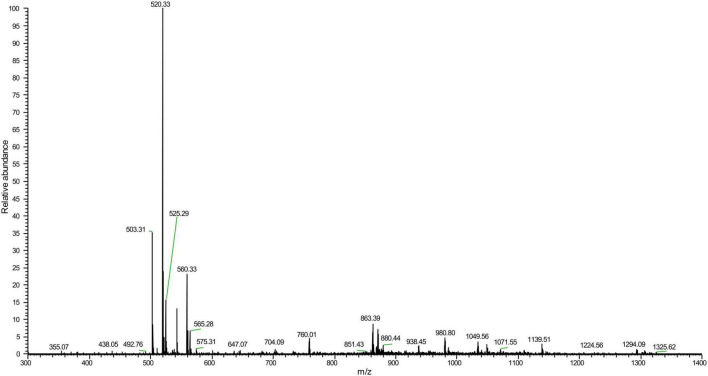
Mass spectrometry of *LFX01*.

Mass spectrometry analysis determined a molecular weight of 1,049.56 Da (Figure 3) and amino acid composition of I-T-G-G-P-A-V-V-H-Q-A of *LFX01* (Supplementary Figure 1). BLAST alignment did not search any similar protein sequence with *LFX01* in protein storage databases.

### Enzyme Sensitivity, Acid-Base, and Thermal Stability

**FIGURE 4:**
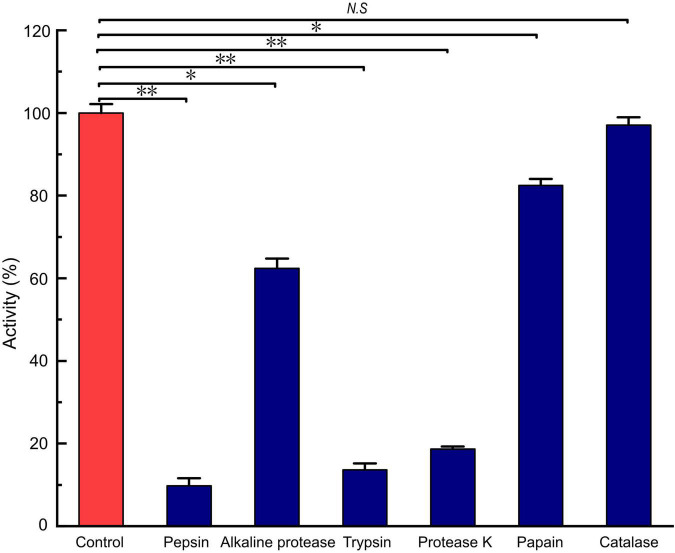
Analysis of enzymes on the stability of *LFX01*. *p < 0.05, ^**^p < 0.01; N.S: no significant difference.

**FIGURE 5:**
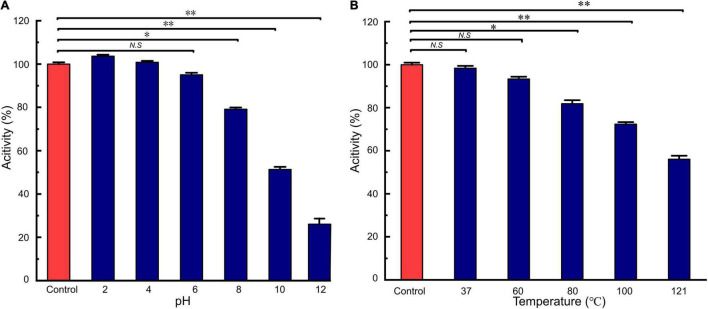
Analysis of acid-base **(A)** and heat **(B)** on the stability of *LFX01.* *p < 0.05, ^**^p < 0.01; *N.S*: no significant difference.

*LFX01* treated with catalase retained 97.02% activity compared to the controls (no significance, *p* > 0.05). This result excluded hydrogen peroxide-based antibacterial activity of *LFX01*. After treated with different proteases, the antibacterial activity of *LFX01* significantly reduced (*p* < 0.05), particularly proteinase K, trypsin, and pepsin (reduced by more than 80%), indicating the sensitivity of *LFX01* to various proteases (Figure 4). Additionally, the antibacterial activity of *LFX01* gradually decreased with increase in pH values (Figure 5A). The antibacterial activity of *LFX01* reached the lowest at pH 12.0 (reduced by 73.74%). The fastest decrease of antibacterial activity of *LFX01* occurred at pH 8∼12 (reduced from 79.16 to 26.26%), and this change in antibacterial activity was significant compared with the controls (*p* < 0.05). Furthermore, the antibacterial activity of *LFX01* gradually decreased with the increase of temperature (37∼121°C) (Figure 5B). Compared with the control, *LFX01* retained 56.06% antibacterial activity at 121°C. The fastest decline in antibacterial activity of *LFX01* occurred at 80∼121°C (reduced from 81.83 to 56.06%).

### Minimum Inhibitory Concentration and Time-Kill Kinetics

**FIGURE 6:**
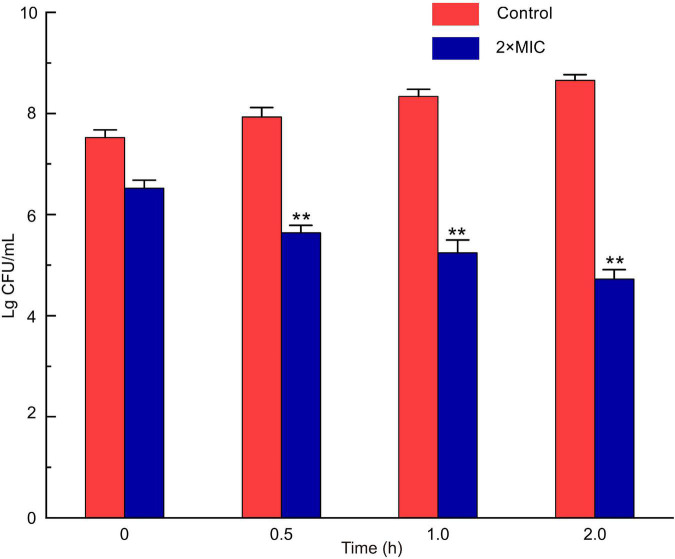
Analysis of time-kill kinetics of *S. flexneri*_14 cells treated with *LFX01.* **p < 0.01; *N.S*: no significant difference.

MIC of *LFX01* against *S. flexneri*_14 was 12.65 μg/mL. The colony number of *S. flexneri*_14 gradually decreased with the treatment time and significantly decreased after 0.5 h (*p* < 0.05) when treated with 2 × MIC for 0∼2 h (Figure 6). The colony number of *S. flexneri*_14 reaches to the lowest value (lg4.65 CFU/mL) at 2 h, confirming the effectiveness of *LFX01* to terminate planktonic *S. flexneri*_14 survival.

### Proliferation and Cell Viability of Planktonic *S. flexneri*_14 Cells

**FIGURE 7:**
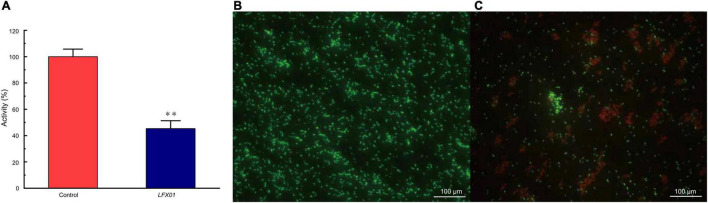
Cell viability of planktonic *S. flexneri*_14 cells after exposure to *LFX01* compared with the untreated control **(A)**. ^**^p < 0.01. Images of planktonic *S. flexneri*_14 cells detected by fluorescence microscopy. **(B)** Control sample and **(C)** *LFX01*-treated *S. flexneri*_14 cells. Live and dead bacteria present green and fluorescence, respectively.

The XTT assays showed that absorbance values of planktonic *S. flexneri*_14 cells was decreased to 45.32% upon exposure to *LFX01* (2 × MIC) for 2 h (*p* < 0.01) (Figure 7A). Compared with the untreated controls (live cells are stained green) (Figure 7B), *S. flexneri*_14 cells died after treatment with *LFX01* (2 × MIC) (red-stained) (Figure 7C). These results further confirmed the effective killing of *LFX01* against planktonic *S. flexneri*_14 cells.

### Antibiofilm Activity of *LFX01*

**FIGURE 8:**
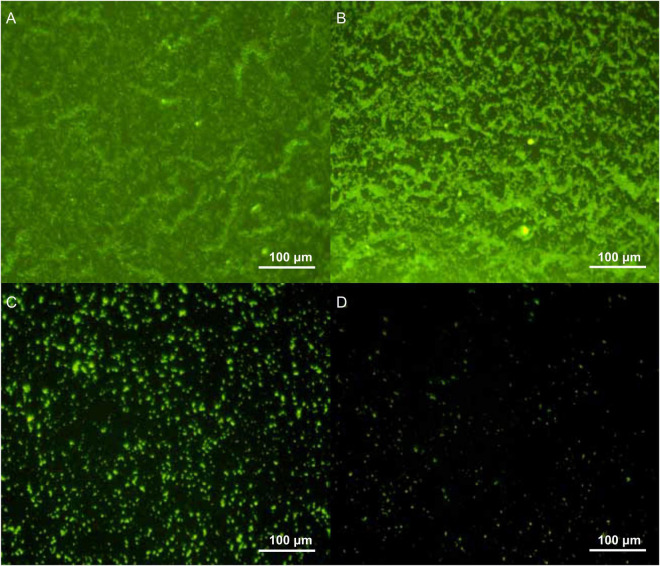
Effect of different concentrations of *LFX01* **(A)**: Control; **(B)**: 1/2 × MIC; **(C)**: 1 × MIC; **(D)**: 2 × MIC) on the biofilm-forming ability of *S. flexneri*_14, as detected by fluorescence microscopy.

**FIGURE 9:**
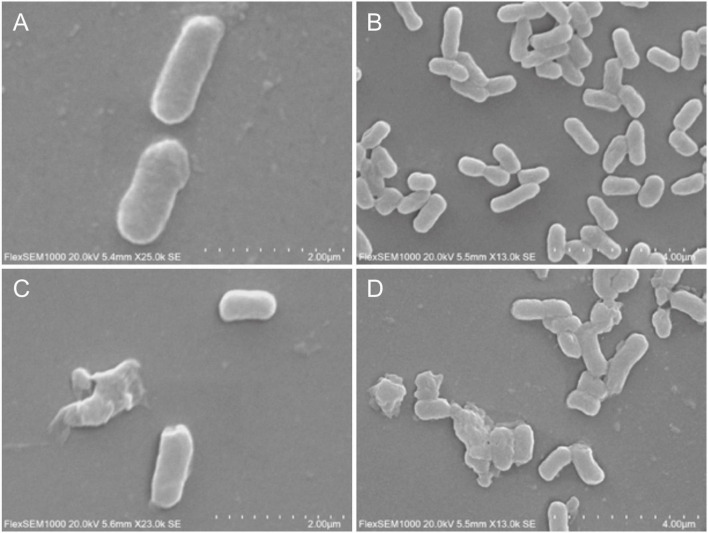
Effect of *LFX01* on biofilm formation of *S. flexneri*_14, detected by SEM. **(A,B)** indicate the control, **(C,D)** indicate treatment.

Biofilm density of *S. flexneri*_14 appeared to be significantly reduced (*p* < 0.05) after treating with *LFX01* at concentrations of 1/2 × MIC, 1 × MIC, and 2 × MIC. Furthermore, quantitative analysis determined that the biofilm exposed to the control (0 × MIC; Figure 8A), 1/2 × MIC (Figure 8B), 1 × MIC (Figure 8C), and 2 × MIC (Figure 8D) of *LFX01* yielded OD595 values of 1.35 ± 0.15, 0.96 ± 0.09, 0.56 ± 0.05, and 0.29 ± 0.03, respectively. *P*-values of all comparison pairs between controls and each experimental group were less than 0.05. The SEM analysis further presented *LFX01*-free *S. flexneri*_14 cells (control) with uniform rod-shaped, smooth surface, neat edge, clear outline, and complete cell structure (Figures 9A,B). However, compared with the control, *S. flexneri*_14 cells incubated with *LFX01* presented uneven shape, wrinkled surface, disruptive cells, content leakage, and cell surface perforations (Figures 9C,D).

## Discussion

To the best of our knowledge, *LFX01* is the first bacteriocin against *S. flexneri*_14 found in fish intestine. Compared with the molecular weight of other LAB bacteriocins reported in previous studies, *LFX01* belongs to a small molecular bacteriocin, as reported in (5592.22 Da from *Lactobacillus pentosus* 31-1) (Zhang et al., 2009), (5383.2 Da from *Lactobacillus salivarius* SMXD51) (Messaoudi et al., 2012), and (1221.074 Da from *L. salivarius* SPW1) (Wayah and Philip, 2018) (665.30 Da from *L. salivarius* CGMCC2070) (Li et al., 2021). Generally, a spatial structure of bacteriocins with smaller molecular weight is more stable under environmental stressors (e.g., temperature, pH, and humidity) (Messaoudi et al., 2012; Wayah and Philip, 2018), implying that *LFX01* can be widely applied to diverse food processing conditions. Additionally, *LFX01* exhibits no homology with other previously reported bacteriocins, indicating that *LFX01* is a novel.

Antibacterial characteristics of bacteriocins are essential parameters of the food industry. The enzyme sensitivity tests excluded the effects of hydrogen peroxide produced by LAB on *S. flexneri*_14 in this study. Meanwhile, *LFX01* sensitivity to proteases indicated that *LFX01* was a bacteriocin possessing protein-like properties (Sung and Jo, 2019). In addition, the antibacterial activity of *LFX01* remained between pH 2 and pH 6, and then significantly decreased by approximately 20, 45, and 74% at pH 8, pH 10, and pH 12, respectively. Previous studies have reported that the antibacterial activity of LAB bacteriocins (produced by *L. salivarius* SPW1, *Lactobacillus fermentum* BZ532, and *Lactobacillus paracasei* HD1-7) significantly reduced by at least 90% when pH exceeded 8 for 2 h (Ge et al., 2016; Baranzan and Koshy, 2018; Rasheed et al., 2020). Accumulating studies have reported that the antibacterial activity of LAB bacteriocins significantly decreased over alkaline conditions (Am et al., 2019; Rasheed et al., 2020). For instance, LAB bacteriocin produced by *Lactococcus lactis* could be completely inactivated by alkali treatment at pH 12 for 2 h (Pmka et al., 2019). In this study, the antibacterial activity of *LFX01* retained 26.26% of the control under pH 12 for 2 h. Additionally, the antibacterial activity of *Lactobacillus rhamnosus* zrx01 bacteriocin and *L. plantarum* B21 bacteriocin significantly reduced by at least 50% when heat treatment exceeded 100°C for 30 min (Golneshin et al., 2020; Zhao et al., 2020). *Lactobacillus acidophilus* NX2-6 bacteriocin exerted 20.00% antibacterial activity of the control under 121°C for 1 h (Meng et al., 2020). In this study, the antibacterial activity of *LFX01* retained approximately 72.32–56.06 % of the control after treatment at 100 and 121°C for 30 min, respectively. Certainly, several previously reported LAB bacteriocins exhibited excellent thermal stability similar to that of *LFX01* (Ge et al., 2016; Pmka et al., 2019; Li et al., 2021). These results suggested that *LFX01* possesses excellent acid-base and temperature tolerance effects. Diversity in the thermal stability and acid-base tolerance of LAB bacteriocins confirms the significant potentiality of LAB bacteriocins as an antibacterial agent in a wide range of temperature and acid-base treatments of food by their combination.

The MIC of *LFX01* against *S. flexneri*_14 assessed in this study was close to or lower than the previously purified LAB bacteriocins (Yi et al., 2018; Wang et al., 2019). For instance, MIC of *XSJ01* against *S. aureus* was 9.85 μg/mL (Li et al., 2021), MIC of bacteriocin *BMP11* against various foodborne pathogenic bacteria (e.g., *S. flexneri*, *L. monocytogenes*, *Cronobacter sakazakii*, and *S. aureus*) was 0.3–38.4 μg/mL (Yi et al., 2018), MIC of bacteriocin *BM1157* against *S. aureus* was 25.0 μg/mL (Yi et al., 2020). This mostly attributed to good killing effects of *LFX01* on planktonic *S. flexneri*_14 cells. Especially, the cell viability of *S. flexneri*_14 significantly reduced after treatment with *LFX01* in the XTT experiments. However, planktonic *Enterococcus faecalis* cells exhibited no reduction with *Pediococcus acidilactici* bacteriocin during XTT assay (Yoon and Kang, 2020). *P. acidilactici* bacteriocin hardly retained the cell viability of *Pseudomonas aeruginosa* cells (Lee et al., 2020). Notably, XTT assay revealed that the cell viability of *S. aureus* cells was reduced by 47% after treating with LAB bacteriocins (produced by *L. salivarius*) (Li et al., 2021). Therefore, LAB bacteriocins presented better reduction effects on cell viability of planktonic foodborne pathogens than non-LAB bacteriocins.

In this study, different MICs of *LFX01* were used to inhibit the formation of *S. flexneri*_14 biofilm. The quantitative analysis showed that *LFX01* could significantly inhibit *S. flexneri*_14 biofilm formation. Further observation revealed that with the increase of MIC concentration, cell density of *S. flexneri*_14 biofilm gradually decreased. Notably, the biofilm formation was effectively reduced at the concentration of 1/2 × MIC, and the biofilm was hardly observed at the concentration of 2 × MIC, indicating an effective concentration of *LFX01* under different antibiofilm requirements. These results confirmed *LFX01* as an effective inhibitor of *S. flexneri*_14 biofilm formation. Therefore, *LFX01* could be further developed to LAB bacteriocin products for inhibiting *S. flexneri*_14 biofilm. Despite these findings, the mechanisms underlying the antibacterial effects of bacteriocins on foodborne pathogens remain indefinite. The SEM observation revealed uneven shape, wrinkled surface, disruptive cells, content leakage, and perforations on the cell surface, which was consistent with the previous study results (Sun et al., 2018; Hossain et al., 2020; Lanhua et al., 2020). Previous studies have concluded that bacteriocins exposure could interfere with DNA metabolism, resulting in cytoplasmic content leakage and decreased activity of key kinases, eventually causing cell death of pathogenic bacteria (Yi et al., 2018; Li et al., 2021; Zhu et al., 2021). Therefore, antibacterial effects of *LFX01* on biofilm formation and activity of planktonic *S. flexneri*_14 cells are primarily attributed to the reduced metabolic activity and perforated cell membrane.

## Conclusion

In summary, a novel bacteriocin *LFX01* produced by *L. plantarum* was purified and characterized for antibacterial activities. *LFX01* exhibited excellent thermal stability and acid-base tolerance. Moreover, the antibacterial activity of *LFX01* against *S. flexneri*_14 showed a reduced rate after treatment with proteases. Furthermore, *LFX01* significantly decreased the cell viability of *S. flexneri*_14 and terminated planktonic bacterial cells. *LFX01* significantly inhibited the *S. flexneri*_14 biofilm formation at low concentrations. Therefore, the antibacterial effects of *LFX01* against *S. flexneri*_14 were attributed to the decreased bactericidal activity and the *S. flexneri*_14 cell membrane injury. This study suggests that *LFX01* could be a promising alternative agent to control *S. flexneri*_14 in foods.

## Data Availability Statement

The datasets presented in this study can be found in online repositories. The names of the repository/repositories and accession number(s) can be found in the article/Supplementary Material.

## Ethics Statement

The experimental protocol was approved by the Ethical Committee of Research of Kunming University of Science and Technology.

## Author Contributions

Q-LZ, X-YD, and L-BL conceived and designed the research. Y-HJ and W-GX conducted the experiments. Q-LZ and X-YD contributed new reagents and analytical tools. Y-HJ, W-GX, and X-YD analyzed the data and wrote the manuscript. X-YD guided the experiments. All authors read and approved the manuscript.

## Conflict of Interest

The authors declare that the research was conducted in the absence of any commercial or financial relationships that could be construed as a potential conflict of interest.

## Publisher’s Note

All claims expressed in this article are solely those of the authors and do not necessarily represent those of their affiliated organizations, or those of the publisher, the editors and the reviewers. Any product that may be evaluated in this article, or claim that may be made by its manufacturer, is not guaranteed or endorsed by the publisher.

## Supplementary Material

Supplementary Figure 1 Analysis of the amino acid composition of *LFX01*.
