# Supplementary figures and images for: A Novel Bacteriocin Against Shigella flexneri From Lactiplantibacillus plantarum Isolated From Tilapia Intestine: Purification, Antibacterial Properties and Antibiofilm Activity
Source: Front Microbiol. 2022 Jan 5;12:779315. doi: 10.3389/fmicb.2021.779315 (PMC8769287; doi:10.3389/fmicb.2021.779315)

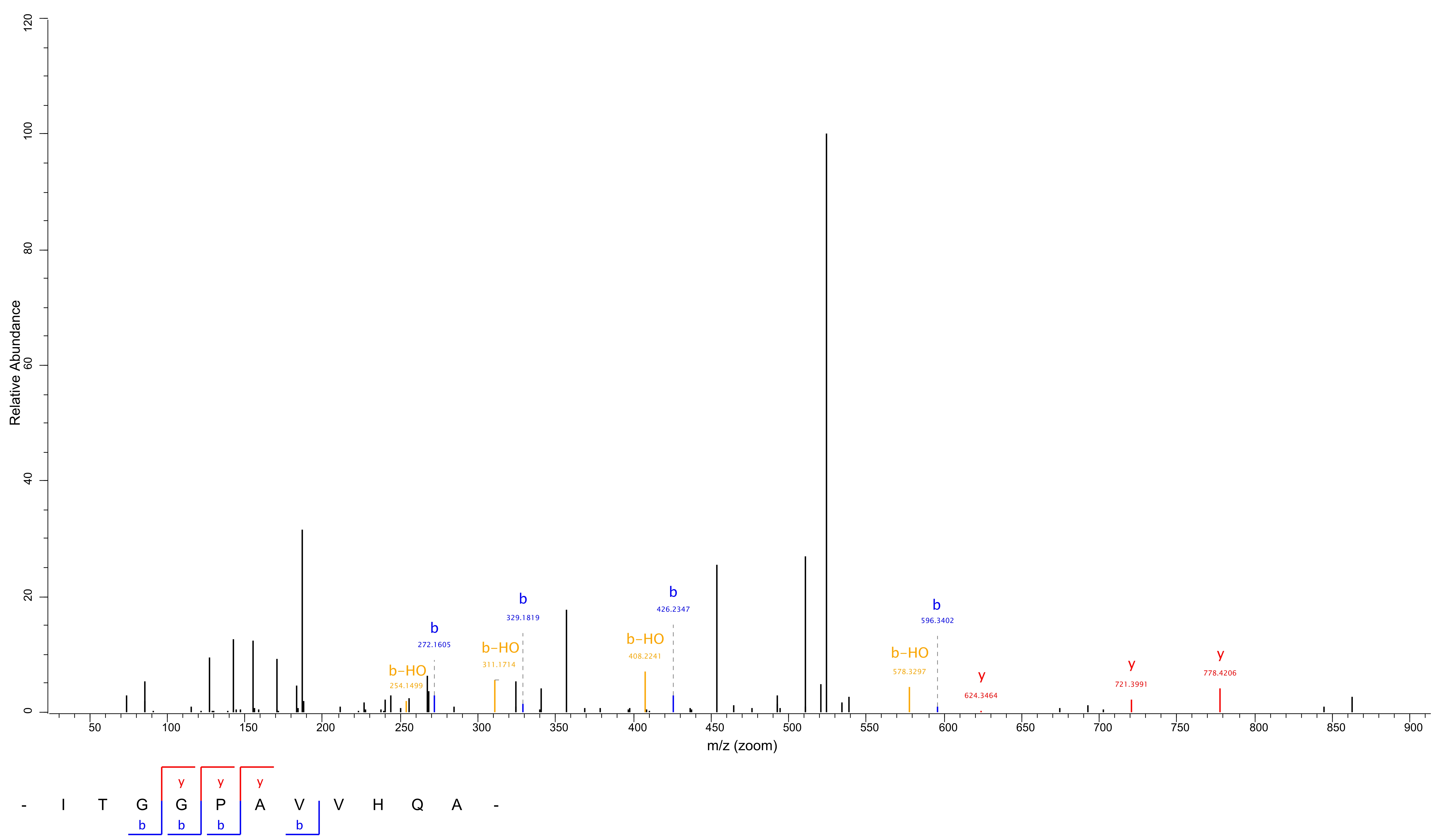

Supplement: Supplementary Figure 1 — Analysis of the amino acid composition of LFX01. [file Image_1.JPEG]
